# The effects of nutrition supplement on rehabilitation for patients with stroke: Analysis based on 16 randomized controlled trials

**DOI:** 10.1097/MD.0000000000029651

**Published:** 2022-09-16

**Authors:** Jianhua Liu, Jige Dong, Jiangzhou Guo

**Affiliations:** a Department of Physical Therapy, Beijing Bo’ai Hospital, Chinese Rehabilitation Research Centre, Beijing, China; b Department of Rehabilitation and Treatment, Wangjing Hospital, Chinese Academy of Traditional Chinese Medicine, Beijing, China.

**Keywords:** function, nutrition, rehabilitation, stroke

## Abstract

Malnutrition is a relatively common and often unrecognized condition in stroke survivors, which may negatively affect functional recovery and survival. Though previous studies have indicated significant role of nutrition supplement for rehabilitation of patients with stroke, the results still remain controversy.

**Objective:**

The present analysis was designed to systematically review effective evidence of nutrition supplement on rehabilitation for patients with stroke.

**Methods:**

A systematic search of PubMed, EMBASE, the Cochrane Library, and Web of Science up to August 1, 2021 was performed to find relevant studies that analyzed the effect of nutrition supplement on rehabilitation of patients with stroke. The primary outcome was functional outcomes and activities of daily living (ADL). The secondary outcomes included disability, all-cause mortality, infections, pneumonia, walking ability, stroke recurrence, and laboratory results indicating nutrition status of patients. All statistical analyses were performed using standard statistical procedures with Review Manager 5.2.

**Results:**

Ultimately, 16 studies including 7547 patients were identified. Our pooled results found no significant difference in total, cognitive and motor FIM score between nutrition supplement and placebo groups, with pooled MDs of 7.64 (95% CI − 1.67 to 16.94; *P* = .11), 0.74 (95% CI − 1.33 to 2.81; *P* = .48), 1.11 (95% CI − 1.68 to 3.90; *P* = .44), respectively. However, our result showed that nutritional interventions had significant effect on ADL for patients with stroke (MD 3.26; 95% CI 0.59 to 5.93; *P* = .02). In addition, nutrition supplement reduced the incidence of infections for patients with stroke, with a pooled RR of 0.65 (95% CI 0.51 to 0.84; *P* = .0008). No significant results were found in disabilities, complication and laboratory outcomes.

**Conclusions:**

The present meta-analysis indicated no statistically significant effect of nutrition supplement on functional outcomes as well as disabilities, complication and laboratory outcomes for patients with stroke. However, it increased ADL and reduced the incidence of infections.

## 1. Introduction

Stroke is one of the leading causes of death in industrialized countries ranging just behind cardiac- and cancer-related causes and is currently the leading cause of disability in Western countries.^[1]^ Stroke incidence varies across Europe between 100 and 700 events per 100,000 inhabitants being highest in Eastern European countries (Lithuania and Poland) and lowest in south Europe (Italy and Spain).^[2]^ The poststroke mortality rate is 20% during the acute period (within 30 days) and 30% at 1 year. Furthermore, stroke is one of most important causes of disability.^[3,4]^ In Italy, about one-third of patients surviving 1 year after a stroke are totally dependent on other people, creating an enormous socioeconomic burden.^[5]^

Malnutrition is frequently observed in patients with stroke, and dysphagia contributes to malnutrition risk.^[6]^ The prevalence of malnutrition after stroke has widely varied from 8% to 34% among published reports. It is estimated that about fifth of patients with acute stroke are malnourished on admission.^[7,8]^ This variation is probably due to patient selection, the definitions of malnutrition, and the method and timing of assessments. In a small study in geriatric patients with severe stroke, 56.3% were malnourished at some point during a hospital stay of > 3 weeks.^[9]^ On the other hand, in poststroke patients residing in an infirmary, the prevalence of malnutrition was 61%.^[10]^

In patients who undergo rehabilitation after ischemic stroke, nutrition strategies are adopted to provide tube-fed individuals with adequate nutrition and/or to avoid the body wasting responsible for poor functional outcome and prolonged stay in the hospital.^[6,9,11]^ From 2000 to 2009, a focus on connections between nutrition and the brain developed with interventions planned to target some brain metabolic alterations primed by acute brain ischemia, such as impaired protein synthesis, excess free radical production, and deficiencies of minerals, including zinc.^[9]^ Investigations have documented that recovery of neurocognitive function in individuals with ischemic stroke may indeed be enhanced by nutrition interventions.^[12–14]^ In addition, the observational data in a large prospective cohort enrolled in the Feed Or Ordinary Diet (FOOD) provide reliable evidence that nutritional status early after stroke is independently associated with long-term outcome.^[15]^

However, though previous studies have indicated significant role of nutrition supplement for rehabilitation of patients with stroke, the results still remain controversy. The present analysis was designed to systematically review effective evidence of nutrition supplement on rehabilitation for patients with stroke.

## 2. Methods and Materials

### 2.1. Search strategy

A systematic search of the PubMed, Cochrane, Web of Science, and Embase were performed up until August 1, 2021; no date restrictions were applied and the reference lists of all relevant articles and reviews were manually searched. One author performed the literature search and another reviewed ambiguous cases. In cases where the authors had a difference of opinion, consensus was reached following discussion. The search, which was restricted to articles published in English and performed according to major Medical Subject Headings (MeSH) or the title and abstract, used the following keywords: (“stroke” OR “poststroke”) AND (“nutrition” OR “malnutrition” OR “protein” OR “amino acid” OR “energy”) AND (“recovery” OR “function” OR “rehabilitation” OR “outcome*” OR “complication*”).

### 2.2. Study selection

Relevant randomized control trials were included in the present analysis if they: (1) patients with stroke who received nutritional supplement during their rehabilitation; (2) studies that comparing intensive nutritional supplementation to routine nutritional supplementation; (3) studies collected and evaluated functional outcomes, activities of daily living (ADL) or other relevant outcomes including disability, all-cause mortality, infections, pneumonia, walking ability, stroke recurrence, length of stay (LOS) and laboratory results.

Studies will be excluded if they meet the following criteria: (1) patients suffered other disease that may influence the outcomes; (2) experimental trials on animals or nonhuman studies; (3) abstracts, letters, editorials, expert opinions, reviews, case reports were excluded; (4) studies without sufficient data or did not meet our including criteria were excluded.

These criteria were used to screen the titles and abstracts of publications to determine whether they were eligible for inclusion. Studies deemed eligible upon title and abstract screening were screened in full-text. Publications were reviewed in duplicate at each stage and discrepancies were resolved by their consensus, or by adjudication by a third reviewer. The present study was approved by the Ethics Committee of Wangjing Hospital.

### 2.3. Quality assessment and data extraction

Quality assessments of the studies were performed independently by 2 researchers and all disagreements were resolved following discussion in conjunction with the input of a third researcher. The previously validated 5-point Jadad scale was used to assess the quality of each RCT.^[16]^ Studies with scores of 0–1 were considered low quality; scores of 2–3 were considered moderate quality; scores of 4 or more were considered high quality. In addition, the risk of bias for each studies and the risk of bias across all studies were evaluated and shown with figures generated by RevMan 5.2 software.^[17]^

Baseline characteristics and outcomes from the included studies were extracted using a standardized extraction form. Key study characteristics included the following data: first author’s name, publication year, country, sample size, mean age and/or age range of the participants, follow-up time, interventions, and main outcomes. Data were extracted by one reviewer and then examined for accuracy and completeness by a second reviewer.

### 2.4. Statistical analysis

Data comparing the outcomes in intervention and control groups were analyzed using standard statistical procedures provided in RevMan 5.2.^[17]^ Dichotomous data were measured with risk ratio (RR) and continuous variable data were measured with mean difference (MD). The heterogeneity between studies was evaluated by the chi-square-based Q statistical test,^[18]^ with *P*_*h*_ value and *I^2^* statistic, ranging from 0% to 100 %, to quantify the effect of heterogeneity. *P*_*h*_ ≤ 0.10 was deemed to represent significant heterogeneity,^[19]^ and pooled estimates were estimated using a random-effect model (the DerSimonian and Laird method^[20]^). On the contrary, if statistical study heterogeneity was not observed (*P*_*h*_ > 0.10), a fixed effects model (the Mantel–Haenszel method^[21]^) was used. The effects of outcome measures were considered to be statistically significant if pooled RRs with 95% CI did not overlap with 1 or pooled MDs with 95% CI did not overlap with 0. Finally, publication bias was assessed by contour-enhanced funnel plots. If the shape of funnel plots revealed no obvious evidence of asymmetry, we considered that there was no obvious publication bias.

## 3. Results

### 3.1. Included studies, study characteristics, and quality assessment

**Figure 1.**
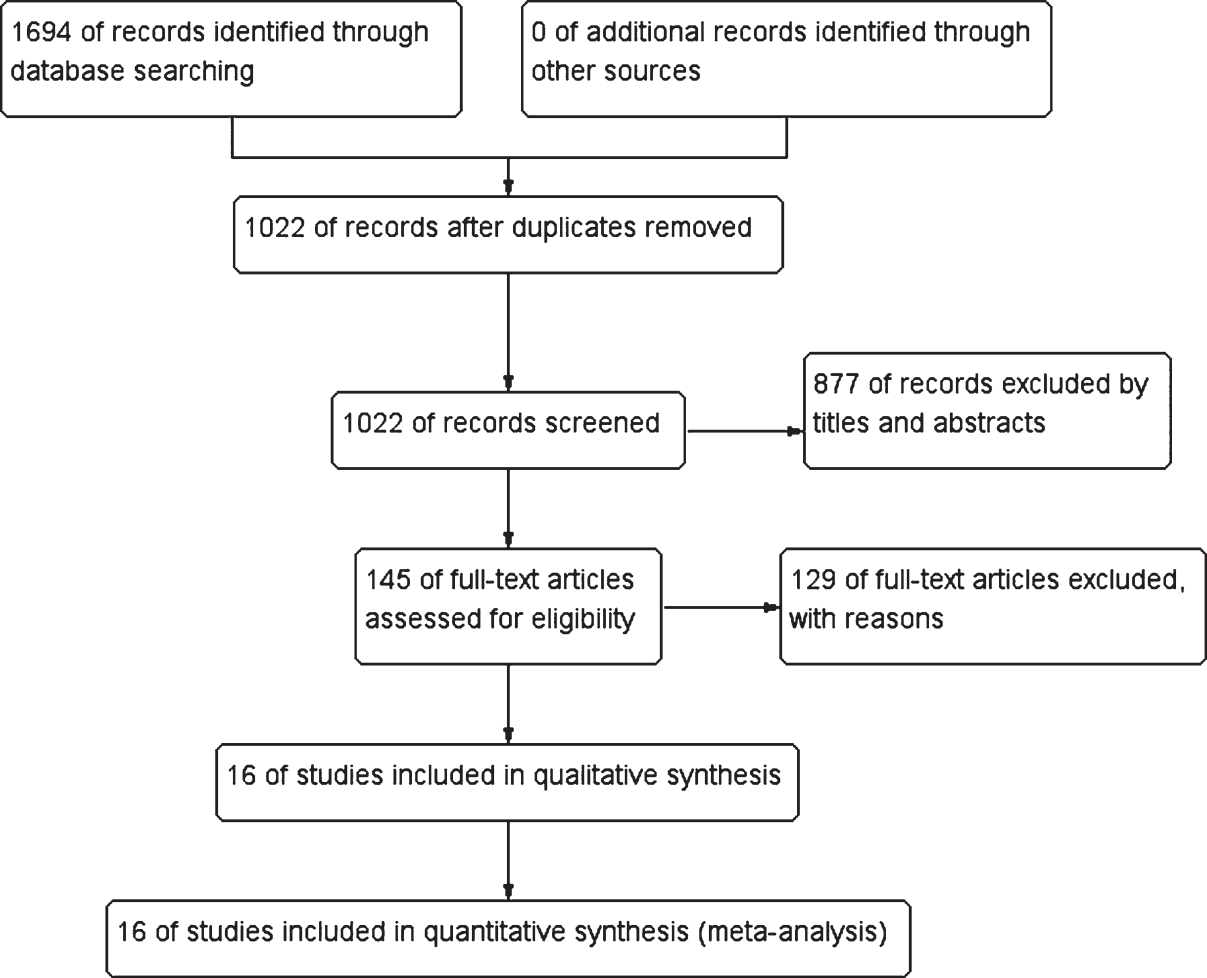
Flow diagram following the PRISMA template of the search strategy for the effects nutrition supplement on the rehabilitation of patients with stroke.

At the beginning of the search, a total of 1964 records of citations were obtained; 1022 of records were reviewed further after duplicates were removed. Via screening the titles and abstracts, 877 studies were excluded preliminarily and then 145 studies were chosen to get full texts for further evaluation. After reading the full texts, 129 studies were excluded further. Eventually, 16 RCTs^[22–37]^ (N = 7547 participants) were included in this systematic review and meta-analysis. Of these studies, 2 were designed as multicenter randomized controlled trials.^[25,26]^ The detailed search process and summary of studies are shown in the study flow diagram (Fig. 1). The other characteristics of each study are shown in Table 1.

**Table 1:** The characteristics of included studies in this meta-analysis.

Study ID	Country	Sample size	Stroke subtype	Age (mean ± SD, range)	Follow-up time	Interventions	Main outcomes
Aquilani R (2014)	Italy	38	Ischemic and hemorrhagic	68 ± 13.2 71.3 ± 10	Discharge	Nutritional mixture supplement that provided 8 g of EAAs/d (4 g in the morning + 4 g in the afternoon diluted in half a glass of water), until discharge	Arterial amino acid concentrations and muscle amino acid arteriovenous difference.
Bellone JA (2019)	USA	16	Ischemic and hemorrhagic	58.13 ± 13.62 59.63 ± 13.48	Discharge	Pomegranate (1 g of a concentrated blend of polyphenols, equivalent to levels in approximately 8 oz of juice (approximately 755 mg of gallic acid equivalents: profile includes ellagitannins, gallotannins, ellagic acid, and flavonoids)) supplement twice per day (morning and night) for 1 week.	Neuropsychological testing (primary outcome: Repeatable Battery for the Assessment of Neuropsychological Status) and functional independence scores
Boselli M (2012)	Italy	136	Ischemic and hemorrhagic	62.7 ± 12.4 63.9 ± 17	60 days	Nutritional mixture supplement with 8 g of EAAs/day (4 g in the morning + 4 g in the afternoon diluted in half a glass of water), 60 days	The incidence of infections, Relationship Between Measured Variables and Functional Independence, Risk-Identifying Variables
Dennis MS (2005a)	Multi-countries	4023	NR	78 ± 10 80 ± 7	6 months	Regular hospital diet plus oral nutritional supplements (360 mL at 6.27 kJ/mL and 62·5 g/L in protein every day), Until discharge	Death or poor outcome (modified Rankin scale [MRS] grade 3–5)
Dennis MS (2005b)	Multi-countries	859	NR	76 ± 11 76 ± 11	6 months	Starting enteral tube feeding (via the clinician’s preferred tube) as soon as possible, for > 7 days	Risk of death
Gariballa SE (1998)	UK	42	Ischemic	78 ± 10 80 ± 7	2, 4, 12 weeks, discharge	Regular hospital diet plus a twice daily oral nutritional supplement of ≥ 400 mL containing 600 kcal, 4 weeks or until death or discharge	Energy and protein intakes, change in nutritional status, disability, infective complications, length of stay, and mortality
Ha L (2010)	Norway	124	Ischemic and hemorrhagic	79.7 ± 6.8 78.5 ± 7.4	3 months	Individualized nutritional care using established oral energy- and protein rich feedings or enteral tube feeding, Until discharge	The percentage of patients with weight loss ≥ 5%; QoL, handgrip strength and length of hospital stay
Irisawa H (2020)	Japan	179	NR	79.7 ± 11.5	4 weeks	NR	Muscle mass and the nutritional status, activities of daily living
Mizushima T (2020)	Japan	668	Ischemic	74 ± 66-80 78 ± 72-84	90 days	NR	Transfer to acute care and death; 2-year mortality; FIM-motor effectiveness
Nishioka S (2017)	Japan	264	NR	78.5 ± 7.5 78.3 ± 7.2	3 weeks	oral protein supplementation of 20 g/d for 21 d	the ability of participants; Achievement of full oral intake, malnutrition risk
Nishioka S (2020a)	Japan	420	Ischemic and hemorrhagic	80.1 ± 8.0 77.2 ± 7.6	NR	NR	Recurrent stroke, prestroke ADL, Total FIM, Comorbidities, Disabilities
Nishioka S (2020b)	Japan	113	Ischemic and hemorrhagic	77 (66.5–84) 77.5 (63.5–82)	3 months	Protein 1.5 g/kg (= 24% energy), carbohydrate 3.12 g/kg (= 50% energy), carbohydrate–protein ratio 2.08, lipids 0.72 g/kg (= 26% energy)	Malnutrition, muscle mass and oral status, and swallowing function recovery
Nishiyama A (2019)	Japan	290	Ischemic and hemorrhagic	76.5 ± 7.5 80.2 ± 7.8	3 weeks	21 days of daily supplementation with a formula providing 20 g of protein and 250 kcal (carbohydrate 28.2 g, lipids 7 g in addition to the 20 g of protein)	ADL was evaluated using FIM, and nutritional status
Rabadi MH (2008)	United States	116	Ischemic and haemorrhagic	75 ± 10.58 73.58 ± 13.02	Discharge	Intensive nutritional supplement every 8 h by mouth (120 ml, 240 calories, 11 g of proteins, 90 mg of vitamin C), Until discharge	Change in total score on the FIM, the FIM motor and cognitive subscores, length of stay, 2-minute and 6-minute timed walk tests
Yoshimura Y (2019)	Japan	113	Ischemic and hemorrhagic	80.8 + 7.1 78.9 + 6.3	90 days	Early nasogastric nutrition using a solution with high nutritional content, 21 days	Physical function, appendicular muscle mass, muscle strength
Zheng T (2015)	China	146	Ischemic and hemorrhagic	71.4 ± 9.3 71.8 ± 10.1	21 days	Either Nutrison fiber, Swiss High (RAE; 4.18–6.27 kJ/ml), or a solution with high nutrition content made by nutritionists and based on condition, body weight, and nutritional status. Energy requirements were in the range of 83.68–125.52 kJ/kg/day	Nutritional status, nosocomial infection, and mortality rates

FIM = functional independence measurement, NR = no report, QoL = quality of life.

**Figure 2.**
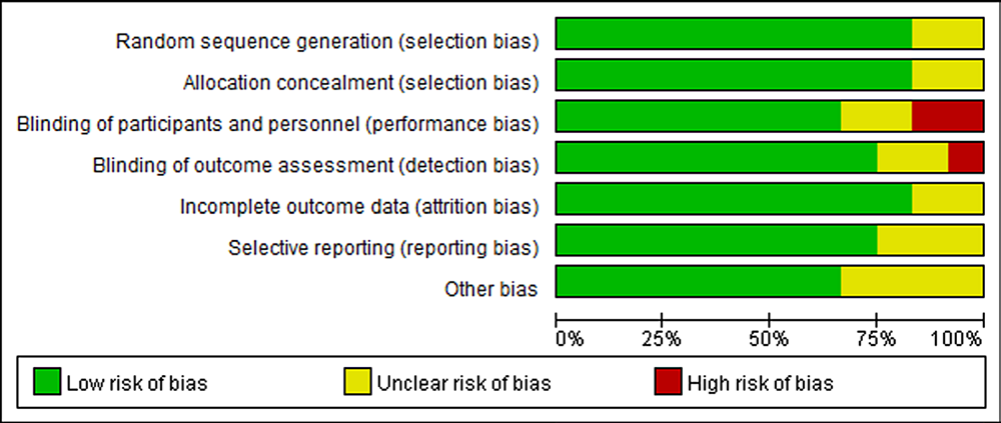
Risk of bias graph: review authors’ judgments about each risk of bias item presented as percentages across all included studies.

**Figure 3.**
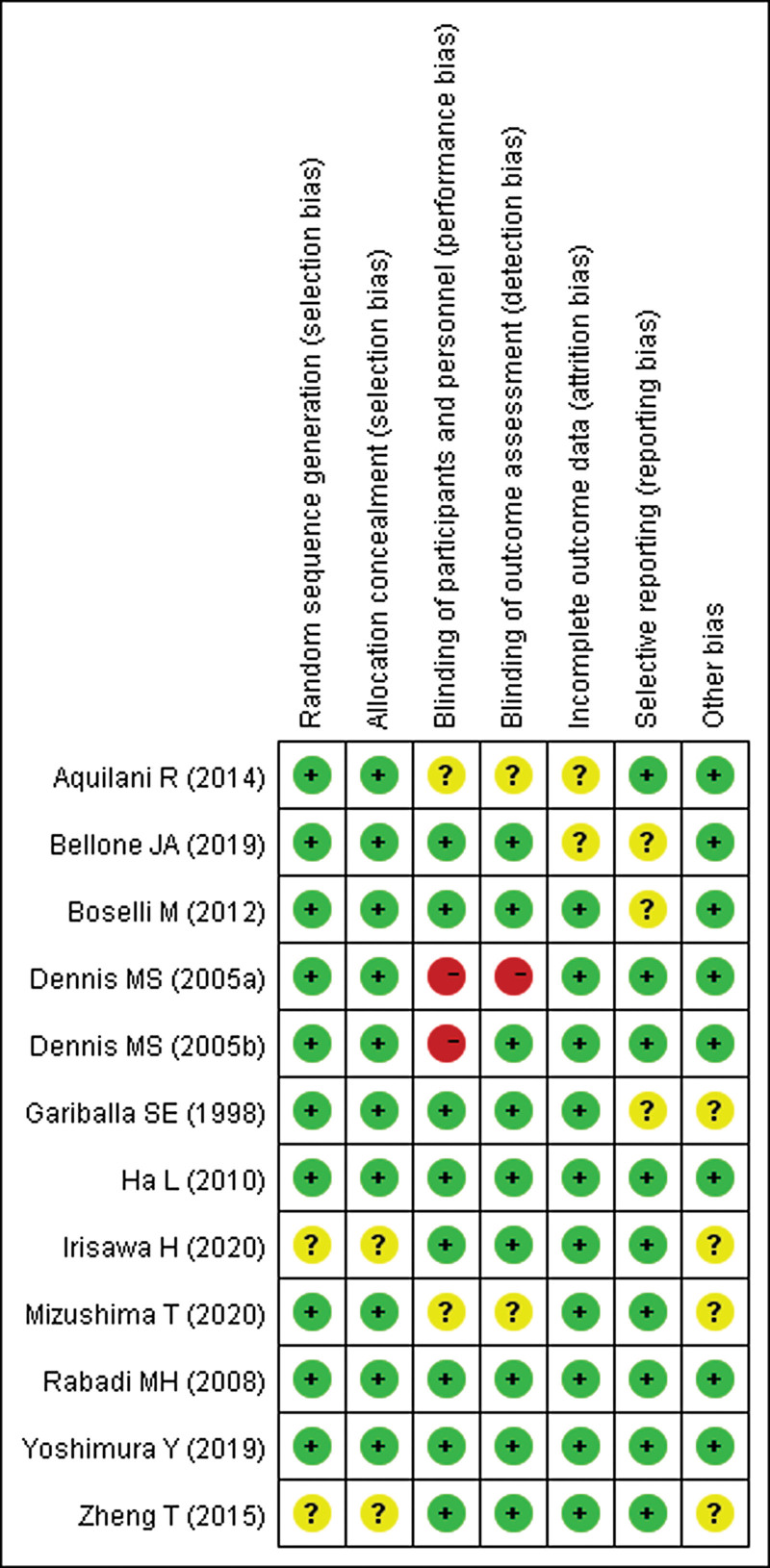
Risk of bias summary: review authors’ judgments about each risk of bias item for each included study.

According to our definitions, there was no low quality studies included in this analysis. Except 4 studies^[22,29,30,37]^ evaluated as moderate quality, the other studies were rated as high quality (75%). Additionally, risk-of-bias graphs were generated to further identify the risk of bias of the including studies. The risk of bias for each RCT was presented as percentages across all included studies, and the risk-of-bias item for each included study was displayed (Figs. 2 and 3). The risk-of-bias graphs indicated generally low risk of selection, performance, attrition, detection, reporting and other bias. High risk of bias was mainly observed in performance and detection bias in 2 studies.^[25,26]^ Unclear risk of bias was dispersedly observed in each items.

### 3.2. The effect of nutrition supplement on FIM score

**Figure 4.**
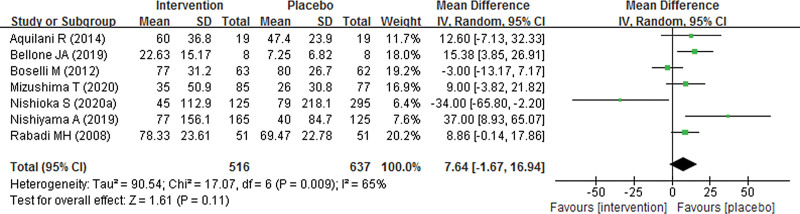
Forest plot of total FIM score of nutrition supplement for patients with stroke.

**Figure 5.**
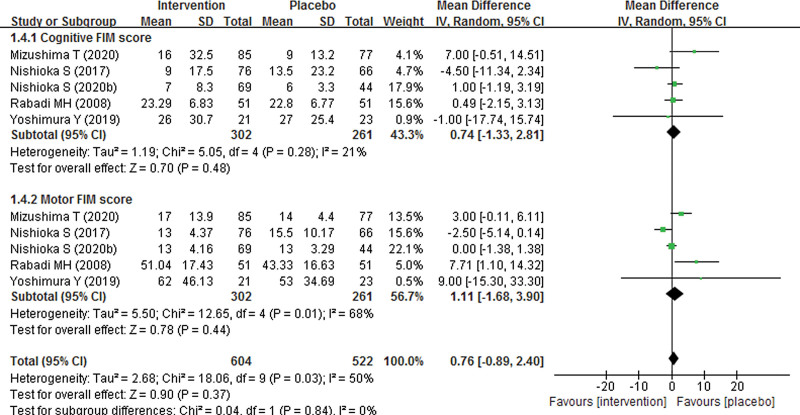
Forest plot of subgroup FIM scores of nutrition supplement for patients with stroke.

Seven studies with 1153 participants were included to assess the effect of nutrition supplement on FIM score. As shown in Figure 4, our pooled result found no significant difference in total FIM score between nutrition supplement and placebo groups, with a pooled MD of 7.64 (95% CI − 1.67 to 16.94; *P* = .11). In addition, we also explored subgroup FIM scores including cognitive and motor FIM score between nutrition supplement and placebo groups. However, the pooled results also showed no significant difference in cognitive and motor FIM score, with pooled MDs of 0.74 (95% CI − 1.33 to 2.81; *P* = .48) and 1.11 (95% CI − 1.68 to 3.90; *P* = .44), respectively (Fig. 5). As significant heterogeneity was found, the pooled results were analyzed using random effect models.

### 3.3. The effect of nutrition supplement on disabilities

**Figure 6.**
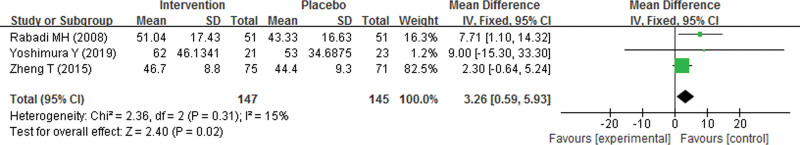
Forest plot of ADL of nutrition supplement for patients with stroke.

Three studies explored the effect of nutrition supplement on ADL of patients with stroke. As Figure 6 shows, the pooled result indicated that nutritional interventions had significant effect on ADL for patients with stroke (MD 3.26; 95% CI 0.59 to 5.93; *P* = .02). As no significant heterogeneity was found (*P* = .31, *I^2^* = 15%), the pooled result was analyzed using random effect model.

In addition, we also explored the effect of nutrition supplement on other disabilities. However, the pooled results indicated no significant difference between nutrition supplement and placebo groups in the incidence of disability (RR 1.08; 95% CI 0.99 to 1.19; *P* = .09), dysphagia (RR 1.59; 95% CI 0.88 to 2.87; *P* = .12), aphasia (RR 1.16; 95% CI 0.92 to 1.45; *P* = .21), neglect (RR 0.68; 95% CI 0.30 to 1.55; *P* = .36), and dysarthria (MD 0.88; 95% CI 0.67 to 1.14; *P* = .33) respectively (Table 2).

**Table 2:** The pooled results of the effect of nutrition supplement on disabilities.

Disabilities	No. of study	Sample size	Pooled results			Analytic effect model
			RR	95% CI	*P* value	
Disability	4	5624	1.08	0.99, 1.19	0.09	Fixed effect model
Dysphagia	2	582	1.59	0.88, 2.87	0.12	Random effect model
Aphasia	2	582	1.16	0.92, 1.45	0.21	Fixed effect model
Neglect	2	162	0.68	0.30, 1.55	0.36	Fixed effect model
Dysarthria	2	170	0.88	0.67, 1.14	0.33	Fixed effect model

### 3.4. The effect of nutrition supplement on complications

**Figure 7.**
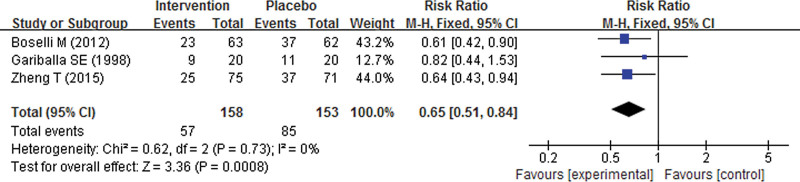
Forest plot of the incidence of infections for patients with stroke.

Our pooled result indicated that patients with stroke receiving nutrition supplement during their rehabilitation experienced significantly lower incidence of infections, with a pooled RR of 0.65 (95% CI 0.51 to 0.84; *P* = .0008) (Fig. 7). However, no significant difference was found in other complications such as mortality (RR 0.90; 95% CI 0.80 to 1.00; *P* = .06), pneumonia (RR 0.97; 95% CI 0.84 to 1.13; *P* = .71) and stroke recurrence (RR 0.94; 95% CI 0.74 to 1.21; *P* = .64) respectively (Table 3).

**Table 3:** The pooled results of the effect of nutrition supplement on complications.

Complications	No. of study	Sample size	Pooled results			Analytic effect model
			RR	95% CI	*P* value	
Mortality	6	5346	0.90	0.80, 1.00	0.06	Fixed effect model
Pneumonia	5	5285	0.97	0.84, 1.13	0.71	Fixed effect model
Stroke recurrence	2	5292	0.94	0.74, 1.21	0.64	Fixed effect model

### 3.5. The effect of nutrition supplement on laboratory outcomes

We evaluated the effect of nutrition supplement on laboratory outcomes. However, our pooled results indicated no significant difference between nutrition supplement and placebo groups in hemoglobin (MD 0.50 g/dL; 95% CI –0.23 to 1.23; *P* = .18), Creatinine (MD –0.04 mg/dL; 95% CI –0.11 to 0.04; *P* = .35), BUN (MD 0.96; 95% CI –1.99 to 3.90; *P* = .52) and albumin (MD –0.04; 95% CI –0.48 to 0.39; *P* = .85), respectively (Table 4).

**Table 4:** The pooled results of the effect of nutrition supplement on laboratory outcomes.

Complications	No. of study	Sample size	Pooled results			Analytic effect model
			MD	95% CI	*P* value	
Hemoglobin (g/dL)	4	453	0.50	–0.23, 1.23	0.18	Random effect model
Creatinine (mg/dL)	3	325	–0.04	–0.11, 0.04	0.35	Fixed effect model
BUN (mg/dL)	2	325	0.96	–1.99, 3.90	0.52	Fixed effect model
Albumin (g/dL)	8	806	–0.04	–0.48, 0.39	0.85	Random effect model

### 3.6. Publication bias

**Figure 8.**
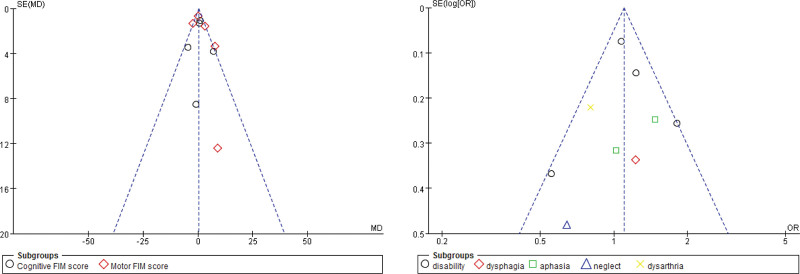
Funnel plot for publication bias of comparison between nutrition supplement and placebo for patients with stroke regarding to FIM score and disability.

Begg funnel plots were generated to assess publication bias in the included studies. As Figure 8 shows, no obvious asymmetry which indicated no clear evidence of publication bias of comparison between nutrition supplement and placebo for patients with stroke regarding to FIM score and disability.

## 4. Discussion and Conclusion

### 4.1. Malnutrition and stroke

Malnutrition is a relatively common and often unrecognized condition in stroke survivors, which may negatively affect functional recovery and survival. Mizushima T (2020) reported the prevalence of moderate and severe malnutrition was 12.7% and 11.5%, respectively.^[30]^ The author also indicated severe malnutrition did not provide additional prognostic information concerning risk of the combined outcome or 2-year mortality. Conversely, severe malnutrition was associated with poorer functional outcome as expressed by FIM-motor effectiveness.^[30]^

The association between nutrition status and rehabilitation outcomes has not been well studied. From 2000 to 2009, a focus on connections between nutrition and the brain developed with interventions planned to target some brain metabolic alterations primed by acute brain ischemia, such as impaired protein synthesis, excess free radical production, and deficiencies of minerals, including zinc.^[8,38]^ Investigations have documented that recovery of neurocognitive function in individuals with ischemic stroke may indeed be enhanced by nutrition interventions. This is particularly important in light of the following considerations^[6]^: (1) no drug acting on damaged brain structures is available in clinical practice for the rehabilitation of patients with ischemic stroke; (2) 80% of the recovery of neurological impairment occurs within the first 30 days after acute ischemia,^[10,39]^ which suggests that every effort should be made to obtain the best functional outcomes during this period of rehabilitation; (3) a large proportion of patients with stroke may have a catabolic state^[40]^; (4) 30 to 35 days after a stroke, patients tend to have the same nutrition deficits as at the time of their admission to a rehabilitation center^[40]^; (5) important calorie-protein deficits have been found 6 months after an acute stroke^[9]^; (6) patients with ischemic stroke have reduced plasma levels of tyrosine, the amino acid precursor of brain adrenergic neurotransmitters (epinephrine, norepinephrine, dopamine).^[41]^

### 4.2. The effect of nutrition supplement on rehabilitation

The present analysis systematically review and assessed the effect of nutrition supplement on rehabilitation for patients with stroke, 16 studies including 7547 patients. Our results supported the points that nutrition supplement could reduce the incidence of infections and could increase ADL of patients with stroke. However, we failed to observed significant effect on function and disability recovery as well as other complications, stroke recurrence, and laboratory results. Nishiyama A (2019)^[34]^ found that FIM score was significantly higher in the high energy intake group compared with the low group (median 113 vs 71, *P* < .001). FIM efficiency was also higher in the high group (median 0.31 vs 0.22, *P* < .001) and thought that high energy intake was associated with higher FIM efficiency.^[34]^ Yoshimura Y (2019) The FIM-M score increased significantly in leucine-enriched amino acid supplement group compared with the control group (*P* < .045).^[36]^

In addition, FOOD Trial Collaboration reliably assessed the importance of baseline nutritional status as an independent. The trial included a total of 3012 patients, and 2955 (98%) were followed up. Of the 275 undernourished patients, 102 (37%) were dead by final follow-up compared with only 445 (20%) of 2194 patients of normal nutritional status (OR 2.32; 95% CI 1.78–3.02). After adjustment for age, prestrike functional state, and stroke severity, this relationship, although weakened, still held (OR 1.82; 95% CI 1.34–2.47). In addition, it was indicated that undernourished patients were more likely to develop pneumonia, other infections, and gastrointestinal bleeding during their hospital admission than other patients. Some of the results were also demonstrated in our pooled results. For example, we found that nutrition supplement could decrease the incidence of infections.

### 4.3. The limitations of this work

This work has several limitations, which need to be taken into account when designing future studies. First, the follow-up time of the studies included in our analysis was much too various, which may lead to any risk bias of our results. However, because of significant heterogeneity in follow-up time of these studies, we failed to conducted subgroup analysis according to follow-up time. Second, the nutrition supplement composition was various. Reports of nutrition supplement for patients with stroke including the antioxidant vitamins E and C, energy, protein and zinc intake. Pellicane AJ (2013) indicated that number of complications, length of stay, and functional outcomes in this patient were not affected by prealbumin levels, protein intake, or calorie intake.^[15]^ However, Boselli M (2012) demonstrated that supplementation with oral essential amino acids (EAAs) may reduce the occurrence of nosocomial infection among patients with brain injury (stroke, trauma, anoxic coma),^[24]^ which was supported by our results. Third, the majority of our included studies included both ischemic and hemorrhagic stroke patients and did not perform subgroup analysis. The authors explained that they included both ischemic and hemorrhagic individuals because, in the rehabilitative phase of stroke, these 2 groups have similar metabolic, nutritional, functional profile. Thus, we failed to conducted subgroup analysis according to stroke subtypes. Fourth, we failed to perform subgroup analysis according to the neurological deficits of the patients as the original studies did not evaluate the outcomes separately according to the neurological deficits of the patients. This was one of our limitations and we suggested future researches could perform this subgroup analysis as more research comes out. Finally, in the present analysis, we did not define the age of trial participants. However, we selected trials in which the participants’ mean age was over 65 years because all the trials had no restriction on age. Considering the majority of participants were old patients, the effect of nutrition supplement may be impaired by their poor intestinal absorptive or metabolic capability. This may be another potential bias of our work.

## 5. Conclusions

The present meta-analysis indicated no statistically significant effect of nutrition supplement on functional outcomes as well as disabilities, complication and laboratory outcomes for patients with stroke. However, it increased ADL and reduced the incidence of infections.

## Author contributions

JH Liu and JG Dong searched the studies for including and extracted the study data. JZ Guo wrote the initial manuscript. All authors assessed the quality of included studies and revised the manuscript.
